# Enhanced Paclitaxel Efficacy to Suppress Triple-Negative Breast Cancer Progression Using Metronomic Chemotherapy with a Controlled Release System of Electrospun Poly-d-l-Lactide-Co-Glycolide (PLGA) Nanofibers

**DOI:** 10.3390/cancers13133350

**Published:** 2021-07-03

**Authors:** Ming-Yi Hsu, Cheng-Hsien Hsieh, Yu-Ting Huang, Sung-Yu Chu, Chien-Ming Chen, Wei-Jiunn Lee, Shih-Jung Liu

**Affiliations:** 1 Department of Medical Imaging and Intervention, Chang Gung Memorial Hospital, Linkou, Taoyuan 33305, Taiwan; m7259@cgmh.org.tw (M.-Y.H.); m7131@cgmh.org.tw (Y.-T.H.); sungyu@cgmh.org.tw (S.-Y.C.); m7146@cgmh.org.tw (C.-M.C.); 2 Department of Mechanical Engineering, Chang Gung University, Taoyuan 33302, Taiwan; 3 Department of Diagnostic Radiology, Chang Gung Memorial Hospital, Keelung 20401, Taiwan; 4 Department of Emergency Medicine, En-Chu-Kong Hospital, New Taipei City 23741, Taiwan; d118107003@tmu.edu.tw; 5 Graduate Institute of Clinical Medicine, College of Medicine, Taipei Medical University, Taipei 11031, Taiwan; 6 Department of Medical Education and Research, Wan Fang Hospital, Taipei Medical University, Taipei 11695, Taiwan; 7 Department of Urology, School of Medicine, College of Medicine, Taipei Medical University, Taipei 11031, Taiwan; 8 Department of Orthopedic Surgery, Bone and Joint Research Center, Chang Gung Memorial Hospital, Linkou, Taoyuan 33305, Taiwan

**Keywords:** metronomic chemotherapy, nanofibers, paclitaxel, PLGA, triple-negative breast cancer

## Abstract

**Simple Summary:**

Treatment of metastatic triple-negative breast cancer (TNBC) relies on chemotherapy. To improve the efficacy of chemotherapy and avoid systemic toxicity, metronomic chemotherapy using continuous administration of low-dose chemotherapy could be a solution. The paclitaxel-loaded PLGA nanofibers allow for continuous and prolonged drug release, which is compatible with the concept of metronomic chemotherapy. The animal study revealed that the strategy successfully inhibited the growth of the primary tumor and distant metastasis without sarcopenia. These data offer new insights into the role of drug-loaded nanofibers in the treatment of metastatic TNBC.

**Abstract:**

Triple-negative breast cancer (TNBC) is highly aggressive and responds poorly to conventional chemotherapy. The challenge of TNBC therapy is to maximize the efficacies of conventional chemotherapeutic agents and reduce their toxicities. Metronomic chemotherapy using continuous low-dose chemotherapy has been proposed as a new treatment option, but this approach is limited by the selection of drugs. To improve antitumor therapeutic effects, we developed electrospun paclitaxel-loaded poly-d-l-lactide-co-glycolide (PLGA) nanofibers as a topical implantable delivery device for controlled drug release and site-specific treatment. The subcutaneously implanted paclitaxel-loaded nanofibrous membrane in mice was compatible with the concept of metronomic chemotherapy; it significantly enhanced antitumor activity, inhibited local tumor growth, constrained distant metastasis, and prolonged survival compared with intraperitoneal paclitaxel injection. Furthermore, under paclitaxel-loaded nanofiber treatment, systemic toxicity was low with a persistent increase in lean body weight in mice; in contrast, body weight decreased in other groups. The paclitaxel-loaded nanofibrous membranes provided sustained drug release and site-specific treatment by directly targeting and changing the tumor microenvironment, resulting in low systemic toxicity and a significant improvement in the therapeutic effect and safety compared with conventional chemotherapy. Thus, metronomic chemotherapy with paclitaxel-loaded nanofibrous membranes offers a promising strategy for the treatment of TNBC.

## 1. Introduction

Triple-negative breast cancer (TNBC) is defined as cancer that lacks the expression of human epidermal growth factor receptor 2, as well as estrogen and progesterone receptors [1,2]. TNBC includes approximately 20% of breast cancers worldwide. TNBC has aggressive behavior and a high risk of recurrence within the first 3–5 years after the completion of adjuvant chemotherapy; its prevalence is higher in younger women [3,4]. However, the clinical success of targeted therapy is low, and most TNBC patients mainly rely on conventional chemotherapy. There are also no prospectively collected data to indicate that combined chemotherapy can improve overall survival, compared with single-agent sequential cytotoxic chemotherapy. Recently, an antibody–drug conjugate, sacituzumab govitecan-hziy, received Federal Drug Administration approval for metastatic TNBC treatment; however, its response rate only reaches 33.3% [5]. To manage TNBC, there is a critical need to maximize the efficacies of existing chemotherapeutic drugs.

Compared with conventional chemotherapy, metronomic chemotherapy (MCT—i.e., continuous low-dose chemotherapy without prolonged drug-free breaks) is reportedly effective in various cancers, including breast cancer [6,7,8,9]. MCT can directly target and inhibit cancer cells and demolish the tumor microenvironment, which is essential for tumor metastasis [10]. MCT is thus considered an alternative chemotherapy method and a new treatment option [11]. However, the selection of chemotoxic drugs is usually limited because peroral administration is much more convenient than other routes. Single-shot medication with long-term effects could be an ideal alternative. In addition to the therapeutic efficacy of chemotherapy, side effects that influence a patient’s physical and mental conditions should also be considered. Recently, sarcopenia has emerged as a new condition that may increase drug toxicity, decrease response to chemotherapy, and adversely affect the prognosis of cancer patients [12,13,14]. Termination of cancer treatment usually results from poor chemotherapy efficacy or patient intolerance to dose-related systemic toxicity. Therefore, minimizing the toxicity of chemotherapy is also of paramount importance.

In recent years, nanotechnology has provided promising approaches to enhance cancer management [15]. Electrospin manufacturing of nanofibers has attracted considerable attention because of its wide application in the biomedical field. Nanofibers made from biodegradable polymers have excellent biocompatibility and biodegradability properties, which make them ideal for chemotherapy [16,17]. Drug-loaded nanofibers can provide sustained release of chemotherapeutic drugs with almost no side effects and are compatible with the concept of MCT. In addition, nanofibers have a high surface-to-volume ratio, are highly porous, and may be employed to manipulate the release profile of drugs. Multiple pharmaceuticals can be directly encapsulated into nanofibrous carriers to offer site-specific drug delivery into target lesions. Using cell line or animal models, drug-loaded nanofibers have been studied for local tumor control in breast cancer, cervical cancer, lung cancer, prostate cancer, glioma, and sarcoma [16,18,19,20,21,22]. However, nanoparticles are more commonly studied for the treatment of metastatic disease compared with nanofibers. Shan et al. [23] used bovine serum albumin to compare drug delivery profiles between nanoparticles and nanofibers; they concluded that nanofibers could produce more sustained drug release, compared with nanoparticles. Furthermore, nanofibers provide additional advantages such as easy formation, tunable morphology with simple parameter control, and high reproducibility. Therefore, it is essential to investigate the efficacy of nanofibers in treating metastatic TNBC [24].

Paclitaxel (PTX) and docetaxel are among the most active agents for metastatic breast cancer. PTX is a promoter of microtubule polymerization and a radio-sensitizing agent. Compared with docetaxel, PTX has a lower myelosuppressive effect and can be used in patients with mild to moderate hepatic dysfunction. However, Cremophor EL is generally used as the solvent because PTX exhibits poor solubility, which places patients at risk of allergic reactions. To maximize the therapeutic effect and minimize the systemic side effects of PTX against TNBC, we prepared PTX-loaded poly(lactic-co-glycolic acid) (PLGA) nanofibrous membranes using an electrospinning method and engrafted the membranes into a mouse model of disease. PLGA is an ideal material for drug targeting and delivery, with the advantages of sustained release time, good biodegradability, and the absence of toxicity and antigenicity [17]. The PLGA-PTX nanofiber nanodelivery system also helped overcome the disadvantages of poor solubility and hypersensitivity related to the use of Cremophor EL as the solvent [25]. In this research, we used the PTX-loaded PLGA nanofibers to treat metastatic TNBC, evaluated the in vitro and in vivo pharmacokinetics of PTX-loaded nanofibers, and investigated whether the nanofibers could help to inhibit the proliferation of cancer cells and reduce systemic toxicity. Electrospun PLGA nanofiber is a well-established drug delivery system for sustained release of both the hydrophobic and hydrophilic compounds, owning a high safety, which is the major consideration for clinical application. Using PTX-loaded nanofiber for local tumor treatment has been reported in various cancers and can be conjugated with other therapy such as brachytherapy to achieve a better result [26,27,28]. However, electrospun polymeric nanofibers are usually considered to be applicable for local chemotherapy [29]. Based on the concept of MCT, this study breaks the prejudgment and provides evidence that the nanofiber membrane can offer more benefit than expected.

## 2. Materials and Methods

### 2.1. Materials

Resomer RG 503 was used as a commercial source of PLGA (Boehringer, Rhein Ingelheim, Germany) with a 50:50 lactide to glycolide ratio and a molecular weight of 33,000 Da. Dimethyl sulfoxide (DMSO), PTX, and 1,1,1,3,3,3-hexafluoro-2-propanol (HFIP) solvents were purchased from Sigma-Aldrich Co. (St. Louis, MO, USA).

Fetal bovine serum, antibiotics (penicillin-streptomycin), trypsin-ethylenediaminetetraacetic acid, trypan blue stain, and all medium additives were obtained from Life Technologies (Gaithersburg, MD, USA). An enhanced chemiluminescence kit was purchased from Amersham (Arlington Heights, IL, USA). An anti-CD31 antibody (sc-376764) was acquired from Santa Cruz Biotechnology (Santa Cruz, CA, USA). Antibodies specific for cleaved-PARP (#5625), PCNA (#2586) and Ki-67 (#9027) were obtained from Cell Signaling Technology (Danvers, MA, USA).

### 2.2. Preparation and Manufacturing of PLGA-PTX Nanofibrous Membranes

PLGA-PTX nanofiber membranes were fabricated using electrospinning with polymer-to-drug ratios of 10:1 and 5:1, respectively. A blank PLGA membrane was also prepared for comparison. The electrospinning equipment consisted of a high-voltage DC power supply, a syringe pump, a needle with an inner diameter of 0.42 mm, a ground electrode, and an aluminum plate.

To fabricate PTX-loaded nanofibers with a polymer/drug ratio of 10:1, PLGA/PTX (720:72 mg) were first mixed with 3 mL HFIP. A syringe pump was then utilized to electrospin the solution at a flow rate of 0.7 mL/h. The distance between the needle tip (22-gauge) and the ground electrode was 15 cm, and the positive voltage was 18 kV. The temperatures and relative humidities were 22 to 24 °C and 44 to 49%, respectively. The nanofiber membrane in a nonwoven form was collected on an aluminum plate. The nanofibrous membrane with a polymer/drug ratio of 5:1 was manufactured in the same manner, except that the polymer-to-drug dosage ratio used was 660:132 mg. After electrospinning, the spun mats were placed in a vacuum oven for 72 h to evaporate the residual solvent.

### 2.3. Characterization of PLGA-PTX Membranes

#### 2.3.1. Scanning Electron Microscope (SEM) Observation and Fourier Transform Infrared Spectroscopy (FTIR) Assay

After gold coating, a Hitachi S3000N SEM (Tokyo, Japan) was used to study the morphology of the nanofibrous membranes. ImageJ software, version 1.53a was used to determine the distribution of nanofiber diameters from SEM images. The infrared spectrum of the drug-loaded membranes was obtained using FTIR analysis and a Bruker Tensor 27 spectrometer at a resolution of 4 cm^−1^ in the absorption mode; in total, 32 scans were collected. The nanofibrous sample was pressed into KBr discs, and the absorption spectrum was recorded in the range of 650 to 4000 cm^−1^.

#### 2.3.2. Water Contact Angle

The hydrophilicity of the PTX-loaded PLGA membrane was evaluated using a contact angle measurement device (First Ten Angstroms Inc., Portsmouth, VA, USA) to measure the water contact angles of the electrospun nanofibers (*n* = 5).

### 2.4. In Vitro Drug Release Study

An in vitro elution method was used to determine the release characteristics of PTX from nanofibers. Phosphate-buffered saline (PBS, pH 7.4) was used as the dissolution medium. Samples (10 mm in width and 20 mm in length) with two polymer/drug ratios (10:1 and 5:1) were placed in individual glass test tubes. Each tube contained 1 mL PBS (0.15 mol/L; pH 7.4) and was incubated at 37 °C. The dissolution medium was collected at 24-h intervals and analyzed, with fresh PBS (1 mL) added after collection. All samples were tested in triplicate for 6 weeks. The concentration of PTX in the buffer was determined by high-performance liquid chromatography (HPLC). HPLC analysis was performed on the Hitachi L-2200^®^ multisolvent delivery system (Hitachi High-Technologies Corporation, Tokyo, Japan), using a SYMMETRY C8, 3.9 cm × 150 mm HPLC column (Waters, Milford, MA, USA). The mobile phase consisted of distilled water and acetonitrile (Sigma-Aldrich, 50/50, *v*/*v*), with a flow rate of 1.0 mL/min, and absorbance was measured at 227 nm. A calibration curve was prepared (correlation coefficient = 0.99993), and the elution product could be accurately identified and quantified with high sensitivity using the HPLC system. The experiment per composition was carried out three times (*n* = 3).

### 2.5. Cell Lines and Cell Culture

The MDA-MB-231 human TNBC cell line was purchased from the American Type Culture Collection (Manassas, VA, USA). Cells were cultured in Dulbecco’s modified Eagle’s medium (Gibco, Gaithersburg, MD, USA) supplemented with 10% fetal bovine serum (Hyclone, Logan, UT, USA) and 1% penicillin-streptomycin-glutamine (Corning, Corning, NY, USA). Cells were grown as adherent monolayer cultures at 37 °C in a 5% CO_2_ and 95% air atmosphere.

### 2.6. In Vivo Study

#### 2.6.1. Orthotopic Xenograft Mouse Model

The study was approved by the Institutional Animal Care and Use Committee of Taipei Medical University (IACUC Approval No.: WAN-LAC-108-006) and conducted in accordance with institutional guidelines. NOD scid gamma (NSG) mice were obtained from the National Laboratory Animal Center of Taiwan, and animal experiments complied with relevant institutional policies. For the orthotopic xenograft breast cancer model, 5 week-old female NSG mice were anesthetized with 3% isoflurane. MDA-MB-231-GFP/luciferase stable cells (5 × 105 cells) were suspended in a 1:1 mixture of PBS and growth factor-reduced Matrigel, then inoculated into the right inguinal mammary fat pad using a 27-gauge needle. After 14 days, the mice were randomly divided into experimental and control groups based on the Xenogen in vivo imaging system (IVIS) bioluminescence imaging (BLI) results, yielding the following four groups to start treatment with similar size tumors.

- Group 1 (DMSO-Ctrl): DMSO solution was administered by weekly intraperitoneal (IP) injection of 100 μL of solvent (10 μL DMSO and 90 μL PBS) for four consecutive weeks.
- Group 2 (DMSO-PTX): Mixed DMSO and PTX solution was administered by weekly IP injection of PTX for 4 consecutive weeks with an equivalent dose of 10 mg/kg (approximately 0.3 mg per mouse) and an injection volume of 100 μL (10 μL of PTX with a concentration of 30 mg/mL and 90 μL of PBS). The total injection dose of PTX was 1.2 mg per mouse (approximately 40 mg/kg).
- Group 3 (PLGA-Ctrl): Blank PLGA nanofibrous membrane was used as the control; it was subcutaneously implanted through a small abdominal incision, which was later closed with 4-0 Vicryl sutures.
- Group 4 (PLGA-PTX): PTX-loaded PLGA nanofibrous membranes with an equivalent PTX dose of 40 mg/kg (approximately 1.2 mg per mouse) were subcutaneously implanted using the same method.

#### 2.6.2. Evaluation of Antitumor Activity

Antitumor activity was evaluated by dividing 20 mice into four groups of five mice each. Weight was measured and recorded weekly, and in vivo tumor images were captured with the IVIS imaging system (Caliper Life Sciences, Alameda, CA, USA) to measure the signal intensity from the GFP/luciferase vector. After 39 days, mice were sacrificed; ex vivo images of excised tumor-bearing tissues were collected in an IVIS-Spectrum system. Tumors were weighed, fixed, sectioned, and stained with CD31 and Ki-67 for immunohistochemistry (IHC).

#### 2.6.3. Evaluation of Survival Rate

The extended survival benefit was evaluated by dividing a second set of 20 mice into four groups and observing them for 70 days. Mice were euthanized if they exhibited inactivity, hunched posture, spiky hair, weight loss > 30%, or tumor size > 1 cm^3^. The survival rate was determined using the Kaplan–Meier method.

#### 2.6.4. BLI

Mice were intraperitoneally injected with D-luciferin at 5 min before imaging (150 mg luciferin/kg body weight) and then anesthetized by exposure to 3% isoflurane before placement in the IVIS Imaging System (Xenogen, Alameda, CA, USA). The acquisition time was 5 s. The radiance efficiency in the whole-animal or organ bioluminescence signal was analyzed by measuring the photon flux (photons/s/cm^2^/sr) using Living Image^®^ software (Xenogen, Alameda, CA, USA).

#### 2.6.5. Pharmacokinetic Study of PTX

In total, 144 female NSG mice with metastatic TNBC were divided into four groups of 36 mice each, then prepared for in vivo pharmacokinetic studies. Tissue specimens with tumors in the 4th mammary fat pad, the fat pad adjacent to the tumor, and peripheral blood samples were obtained at 12 time points (6, 12, and 24 h, as well as 2, 3, 5, 7, 14, 21, 28, 35, and 42 days) from 3 mice at each time point; samples were stored at −20 °C until analysis by HPLC.

#### 2.6.6. Sample Preparation

Methanol with 0.1% (*v*/*v*) acetic acid was used as the solvent for tissue extraction to avoid rapid destruction of PTX [30]. The tissue samples were homogenized with 3 mL methanol solvent at a speed of 6 m/s for 30 s (Homogenizer Prep-CB24, MedClub, Taoyuan, Taiwan). This process was repeated three times, followed by sample filtration using a polyvinylidene difluoride syringe filter (0.22-μm pore size). After centrifugation at 10,000 rpm for 10 min, the supernatant was collected and injected into the HPLC system for further analysis.

### 2.7. IHC Staining

Resected tumors were fixed in 10% (*v*/*v*) formaldehyde in PBS, embedded in paraffin, and cut into 3-μm sections. Antigen unmasking of paraffin sections was performed in a microwave using 0.1 M citric acid buffer (pH 6.0), and endogenous peroxidase activity was quenched with 3% hydrogen peroxide. Sections were blocked using 5% normal goat serum and then incubated overnight at 4 °C with the anti-CD31 primary antibody (1:100, Santa Cruz, sc-376764) and Ki67 (1:100, Cell Signaling, #9027). The corresponding secondary antibody was then added to the slides and incubated at room temperature for 1 h. The sections were observed under a Zeiss Axiophot microscope (Carl Zeiss Microimaging, Gottingen, Germany); a SPOT digital microscope camera was used to capture the images after incubation with a diaminobenzidine reagent (Boster, Wuhan, China).

### 2.8. Preparation of Total Cell Extracts and Western Blot Analysis

For the tumor tissue, the tissue was dissected and disrupted in liquid nitrogen, then lysed with lysis buffer to extract protein. The total protein concentration in the resulting lysate was determined using the bicinchoninic acid protein assay (Merck, Darmstadt, Germany). Appropriate quantities of protein were resolved in the lane for sodium dodecyl sulphate-polyacrylamide gel electrophoresis (SDS-PAGE) and transferred onto nitrocellulose membranes. The membranes were blocked with 5% skim milk and then incubated overnight with indicated primary antibodies, followed by incubation with the corresponding horseradish-peroxidase-conjugated secondary antibodies (1:5000; Abcam, Cambridge, UK). After washing, the bound secondary antibody was then detected using the Western blotting reagent ECL system (Pierce Biotechnology, Rockford, IL, USA). The original western blot can be found in Figure S1. 

### 2.9. Statistical Analysis

Commercially available SPSS software (version 12.0; SPSS Inc., Chicago, IL, USA) was used to analyze the sample data with paired sample *t*-tests. All data in this study are expressed as means ± standard deviations. *p*-values < 0.05 were considered to indicate statistical significance.

## 3. Results

### 3.1. Preparation and Characterization of PLGA-PTX Nanofibrous Membranes

**Figure 1:**
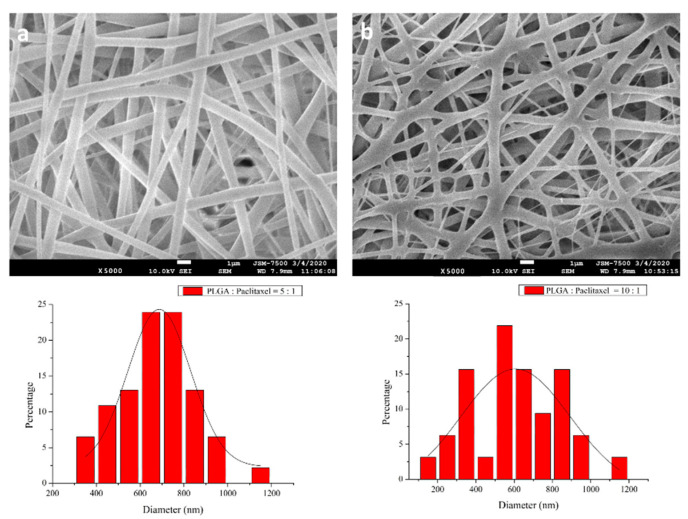
SEM images and diameter distributions of nanofibers with (**a**) 10:1 and (**b**) 5:1 polymer-to-drug ratios (magnification, 5000×, scale bar: 1 µm).

Nanofibrous membranes were successfully produced by electrospinning. Figure 1 shows SEM images of the electrospun PLGA-PTX nanofibers (10:1 and 5:1) at 5000× magnification. The diameters were 650.0 ± 296.0 nm (Figure 1a) and 750.0 ± 236.8 nm (Figure 1b), respectively, for the 10:1 and 5:1 PLGA-PTX nanofibers. All membranes exhibited high porosity.

#### 3.1.1. FTIR

**Figure 2:**
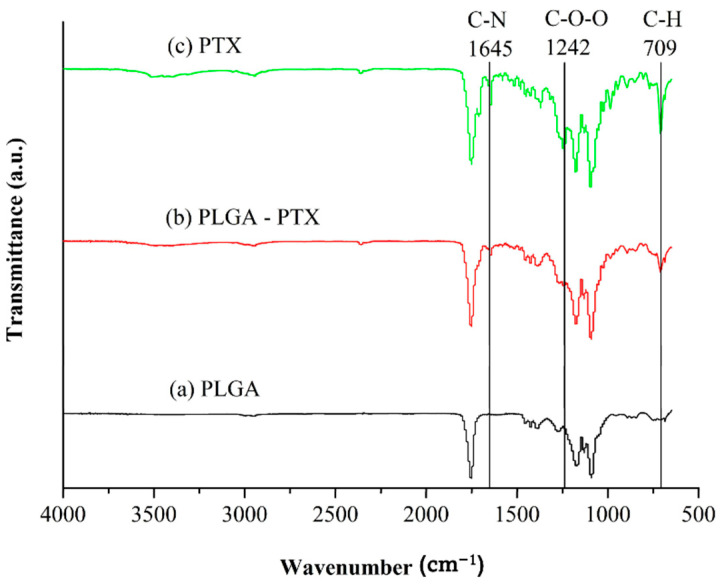
FTIR spectra of (**a**) PLGA, (**b**) PLGA-PTX nanofibers, and (**c**) pure PTX.

Figure 2 demonstrates the FTIR spectra of blank PLGA, PLGA-PTX polymer, and pure PTX. The differences in absorption from blank PLGA at wavenumbers 709 cm^−1^, 1242 cm^−1^, and 1645 cm^−1^ may correspond to the aromatic C-H, C-O-O bending vibrations, and amide N-H of incorporated PTX, respectively [31]. Spectral comparative analysis shows the PTX characteristic peaks range were observed in PTX-loaded nanofiber, which indicates the presence of PTX in the nanofiber formulation. The intensity of the peaks in formulated nanofiber is decreased, indicating the entrapment of PTX in the formulated nanofiber and the formation of amorphous composites between PTX and polymer in the nanofiber formulation.

#### 3.1.2. Water Contact Angles of Nanofibers

**Figure 3:**
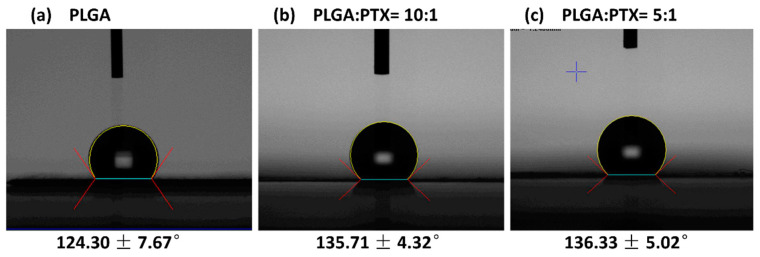
Water contact angles (**a**) 124.30 ± 7.67°, (**b**) 135.71 ± 4.32°, and (**c**) 136.33 ± 5.02°. The drug-eluting membranes showed greater water contact angles compared with the blank PLGA membrane, indicating hydrophilicity reduction after the addition of hydrophobic PTX into the PLGA.

Figure 3 shows that the water contact angle increased with increasing PTX content in the membranes. The contact angles were 124.30 ± 7.67°, 135.71 ± 4.32°, and 136.33 ± 5.02°, respectively, for the blank PLGA, 10:1 polymer-to-drug ratio nanofibers, and 5:1 polymer-to-drug ratio nanofibers. Regardless of the composition, the water contact angles of all samples were >90°, implying high hydrophobicity of these nanofibers. PLGA is known to be a hydrophobic polymer. PTX is also a poorly water-soluble drug, and its presence did not improve the hydrophilicity of electrospun nanofibers.

### 3.2. In Vitro Drug Release of PTX

**Figure 4:**
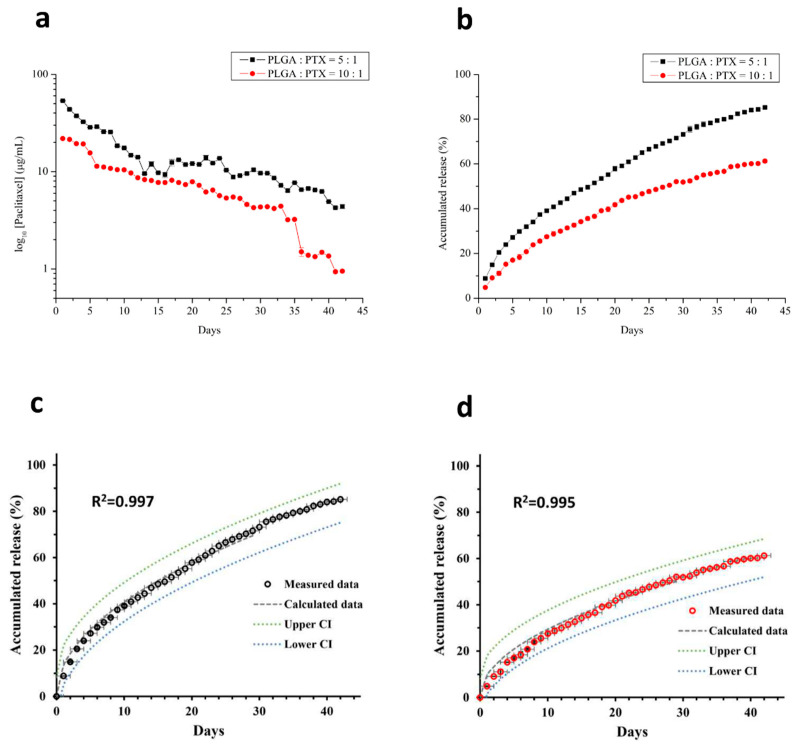
Daily (**a**) and accumulated release (**b**) of PLGA-PTX nanofibers in vitro. The drug release was initially higher in both groups with a gradually decreased release pattern. After 42 days, the accumulated releases of PTX were 85.19 ± 0.4% in the 5:1 polymer-to-drug ratio group and 60.73 ± 1.09% in the 10:1 group. Dissolution profiles and the nonlinear, Higuchi-kinetic-model-calculated release curves of 5:1 (**c**) and 10:1 (**d**) PLGA/PTX ratio nanofibers, with a good fitness indicated by the value of R^2^. All data points are plotted as mean ± SD (*n* = 3). CI—confidence interval.

Figure 4 shows the gradually decreasing in vitro release of PTX from nanofibers. All drug-eluting nanofibers released PTX for 42 days. A comparison of daily PTX release from nanofibers demonstrated higher values for nanofibers with a polymer-to-drug ratio of 5:1. After the 6-week experiment, PTX release values were 85.19% and 60.73% of the total encapsulated PTX for nanofibers with polymer-to-drug ratios of 5:1 and 10:1, respectively. Data derived from the in vitro release were fitted into six common kinetic models including first-order kinetics, second-order kinetics, Higuchi’s plots, Weibull plots, Korsmeyer–Peppas plots, and Hopfenberg plots [29,32] to predict the mechanism of drug release from the formulated nanofiber. The correlation coefficient (R^2^) value was calculated for each model and each experiment was carried out in triplicate. The two PTX nanofibers formulations release kinetics data showed a good fit to the Higuchi square root model, with a high degree of correlation coefficient (R^2^ = 0.9972 and 0.9953 for 1:5 and 1:10 drug to polymer ratio, respectively), which implied the release of PTX from the nanofiber matrix as being a square root of time-dependent process and diffusion controlled [33].

#### 3.2.1. Electrospun PLGA Nanofibers Enhanced the Tumor Growth Inhibitory Effects of PTX in TNBC

**Figure 5:**
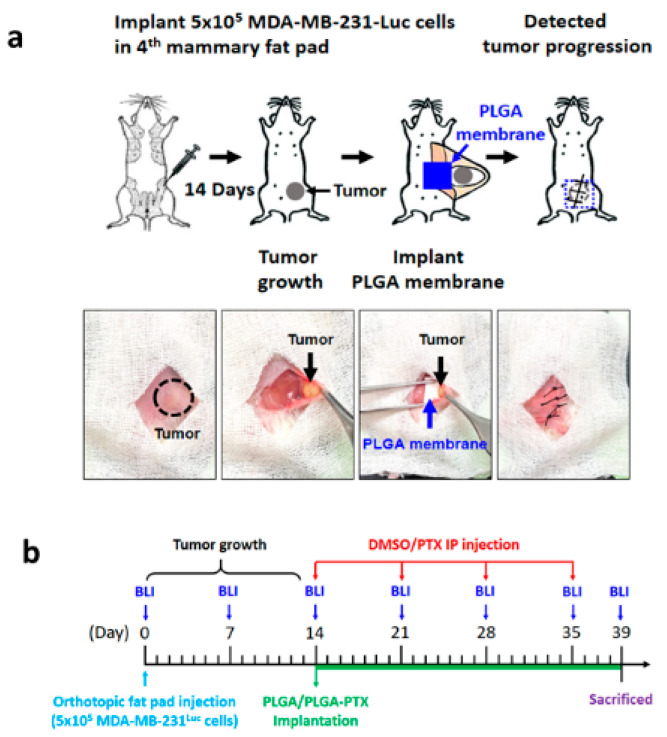
Illustration of the orthotopic breast cancer mouse model and process of drug administration. (**a**) Schematic picture of the mammary gland transplantation procedure. MDA-MB-231-Luc cells were inoculated into the inguinal mammary fat pads of 5 week-old female NSG mice. (**b**) On day 14 after inoculation, mice received either 10% DMSO or PTX in 10% DMSO by IP injection once weekly. In nanofibrous membrane implantation, a 5-mm incision in the epithelium from the gland was made and transplantation of the blank PLGA or PLGA-PTX nanofibrous membrane into the fat pad was carried out. Tumor size and growth were monitored weekly by bioluminescent imaging.

We evaluated the therapeutic efficacy of PTX-loaded PLGA on the establishment and progression of metastatic TNBC cells by inoculating luciferase-expressing human MDA-MB-231-Luc cells within the 4th mammary fat pads of 5-week-old female NSG mice (Figure 5a). After 14 days, mice received one of four treatments: (1) weekly IP injection of 10% DMSO in PBS, (2) weekly IP injection of 10 mg/kg PTX in 10% DMSO, (3) transplantation with PLGA nanofibrous membrane, or (4) transplantation with 40 mg/kg of PLGA-PTX nanofibrous membrane, as illustrated in Figure 5b.

**Figure 6:**
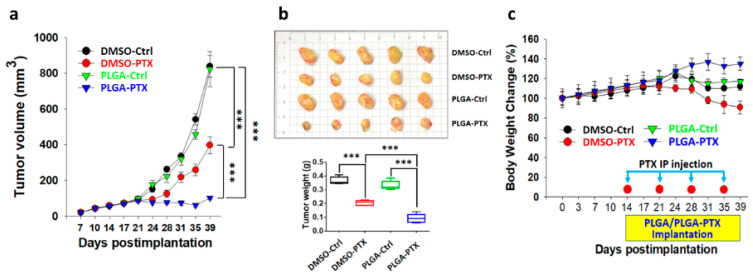
PTX-loaded PLGA nanofibrous membranes could enhance antitumor efficacy in vivo. (**a**) Tumor growth curve. The widths and lengths of MDA-MB-231-Luc tumors were measured, then tumor volumes were calculated using the following formula: volume = width^2^ × length × 0.5236. Data are presented as tumor volumes in each group (*n* = 5 tumors/group). (**b**) Tumors were excised and photographed from MDA-MB-231 xenografts after 39 days (upper panel), and the mean tumor weights of different treatment groups are shown (lower panel). (**c**) Weight changes of the mice in each group. Data are expressed as mean ± SD. *** *p* < 0.001. DMSO-Ctrl, DMSO control; DMSO-PTX, free-paclitaxel in DMSO; PLGA-Ctrl, PLGA nanofibrous membrane only; PLGA-PTX, paclitaxel-loaded PLGA membranes.

Mice were treated with DMSO-Ctrl, free PTX, blank PLGA, and PTX-loaded PLGA. Transplantation of blank PLGA nanofibrous membrane did not affect tumor growth, which was similar to growth after DMSO-Ctrl treatments. Compared with the rapid tumor growth rates in the DMSO-Ctrl and PLGA-Ctrl treatment groups, tumor growth was significantly lower in the free PTX treatment group. In contrast, the PTX-loaded PLGA treatment group had more pronounced reductions of tumor volume and tumor weight of breast cancer at the primary site (Figure 6a,b). The primary tumor at the 4th mammary gland exhibited substantial suppression related to the continuous release of PTX from nanofibers.

The change in body weight is an indicator of systemic toxicity during anticancer drug treatment. As depicted in Figure 6c, the weight assessments revealed a similar slight weight reduction at 2 weeks (day 28) after initiation of either DMSO-Ctrl or PLGA-Ctrl treatment. In comparison with the tumor volume, the weight losses in these two groups might be attributed to cachexia at this high tumor load. Greater weight loss was observed in mice managed with DMSO-PTX, which indicates the systemic toxicity of PTX in mice. However, the PLGA-PTX group showed a continuous and slow increase in body weight, suggesting that the use of PTX-loaded PLGA for direct site-specific tumor treatment could reduce the systemic toxicity of PTX and enhance its antitumor efficacy.

#### 3.2.2. PTX-Loaded PLGA Nanofibrous Membranes Could Effectively Inhibit TNBC Metastasis and Tumor Progression

**Figure 7:**
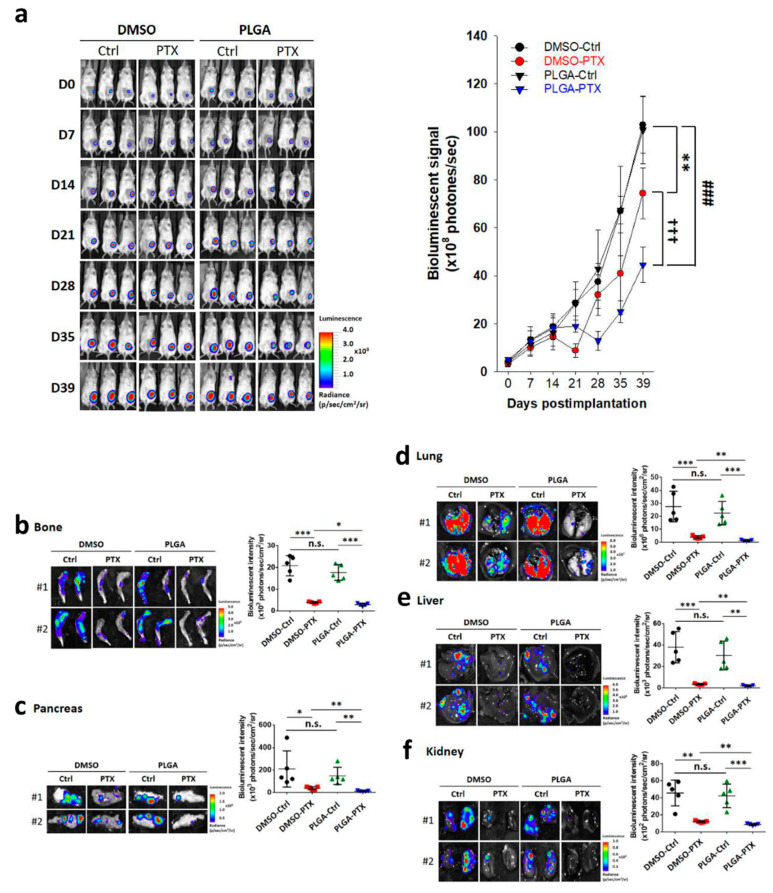
PTX-loaded PLGA nanofibrous membranes could enhance inhibition of tumor growth and metastasis in an orthotopic breast cancer model. (**a**) All mice were euthanized and dissected at 39 days, and representative noninvasive BLI examinations of tumor-bearing mice at indicated times were captured with the IVIS (left panel). Quantitative analysis using the Xenogen imaging signal intensity (photons/s) was performed at the indicated times. Data are expressed as mean ± SD. ** *p* < 0.01, ^###^ *p* < 0.001, ^†††^ *p* < 0.001. (**b**–**f**) Representative ex vivo BLI examinations of metastasis at the end of the MDA-MB-231-Luc orthotopic breast cancer spontaneous metastasis model. Bone (**b**), pancreas (**c**), lung (**d**), liver (**e**), and kidney (**f**) were isolated and imaged with bioluminescence at the end of the study; the mean signal for each group is indicated. Data are expressed as mean ± SD. * *p* < 0.05, ** *p* < 0.01, *** *p* < 0.001.

Consistent with the tumor volume results, the free PTX treatment group exhibited attenuated bioluminescence signal intensity compared with the rapid and significantly higher signals in the DMSO-Ctrl-and PLGA-Ctrl-treated groups. However, the PTX-loaded PLGA group showed much lower photon intensity in mice (Figure 7a). We also conducted ex vivo imaging to avoid the possibility of missed metastasis in whole-body imaging due to inappropriate BLI signal intensity. After the mice had been sacrificed at 39 days, the organs were similarly dissected to observe organ metastasis by BLI. Ex vivo imaging of various tissues indicated that the free PTX treatment group showed significantly lower intensity in organs, including the bone, pancreas, lung, liver, and kidney, compared with the DMSO-Ctrl- and PLGA-Ctrl-treated groups. In contrast, the PTX-loaded PLGA group exhibited a much lower photon intensity (Figure 7b–f). These data suggest that PLGA nanofibrous membranes alone could not inhibit breast tumor growth and metastasis, while PTX-loaded PLGA exhibited greater therapeutic efficacy against breast tumor progression compared with free PTX.

#### 3.2.3. Direct Site-Specific Treatment of Breast Cancer by PTX-Loaded PLGA Nanofibrous Membranes Suppresses Angiogenesis

**Figure 8:**
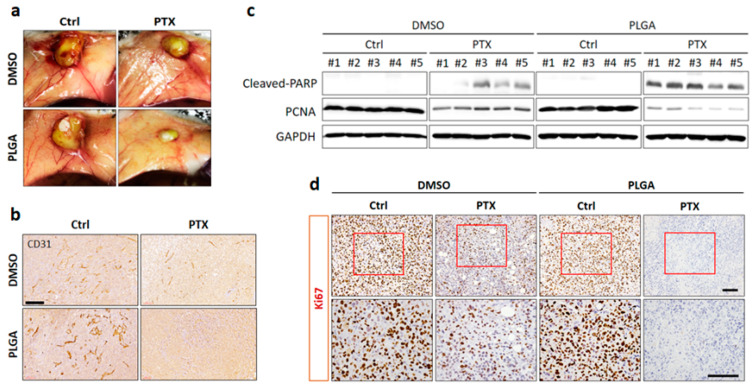
Effect of PTX-loaded PLGA nanofibrous membranes on tumor angiogenesis, cancer cell apoptosis, and proliferation in a breast cancer orthotopic mouse model. (**a**) Tumor size and vascular profile were evaluated by macroscopic observation. (**b**) IHC for CD31 in each treatment group of tumor sections (magnification, 20×). Scale bar, 200 µm. (**c**) Representative western blots for cleaved-PARP and PCNA detection. GAPDH was used as control for equal protein loading. (**d**) IHC analysis of sections from each treatment group of tumor samples for Ki67 (upper panel, magnification, 20×). Red boxes indicate the enlarged area (lower panel, magnification, 40×). Scale bar, 100 µm.

The effects of PTX-loaded PLGA nanofibrous membranes on tumor vasculature were determined by IHC staining for CD31. Macroscopic examination of tumors showed reduced vascularization in the free PTX treatment group compared with the DMSO-Ctrl- and PLGA-Ctrl-treated groups (Figure 8a). Blood vessel staining with anti-CD31 antibody confirmed decreased angiogenesis in the free PTX treatment group (Figure 8b). Importantly, we found that PTX-PLGA treatment further reduced the tumor size and angiogenesis compared with free PTX treatment (Figure 8a,b). Next, to further explore whether induction of apoptosis and suppression of cell proliferation were also involved in PTX-loaded PLGA nanofibrous membranes of tumor suppression effects, an additional apoptotic marker (cleavage of PARP) and proliferative marker (PCNA) were investigated in tumor tissues. By comparison, the results from the Western blot analysis of tumor tissues indicated that the DMSO-Ctrl- and PLGA-Ctrl-treated groups did not cause PARP cleavage, while the free PTX treatment showed markedly upregulated of cleaved PARP levels, which were further increased in PLGA-PTX treatment (Figure 8c and Figure S1). In addition, high levels of PCNA were detected in the DMSO-Ctrl- and PLGA-Ctrl-treated groups and treatment with free PTX led to a significantly reduced expression of PCNA, whereas a more notable decrease was observed upon PTX-loaded PLGA group (Figure 8c). Consistently, in ki67 IHC studies, the DMSO-Ctrl- and PLGA-Ctrl-treated groups showed a higher optical density and free PTX treatment had less anti-Ki67 immunostaining, while PLGA-PTX treatment showed no visually apparent Ki67 staining (Figure 8d). These results illustrated that the antitumor effects of PTX-loaded PLGA could not only suppress angiogenesis in tumor microenvironment but also directly induce apoptosis and inhibit cancer cell proliferation of cancer cells.

#### 3.2.4. PLGA-PTX Transplantation Contributed to Prolonged Overall Survival Rates

**Figure 9:**
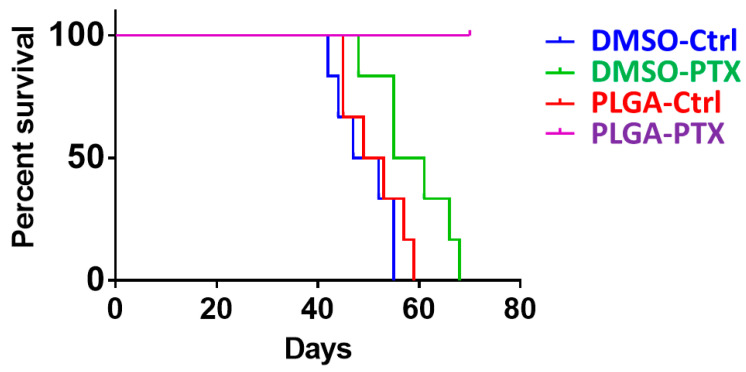
PLGA-PTX improved the survival rate in orthotopic xenograft tumor models. Kaplan–Meier survival analysis, beginning at 2 weeks after tumor cell injection (day 0) and continuing until death. Death was recorded when an animal met predetermined euthanasia criteria. Higher survival was observed among mice in the PLGA-PTX group than among mice in other groups. Moreover, no mice had died in the PLGA-PTX group by the end of the experiment, indicating a greater survival benefit.

Our previous data demonstrated that PLGA-PTX treatment markedly reduced tumor growth and metastasis in an orthotopic breast cancer mouse model. Next, we evaluated the general therapeutic effect on the basis of overall survival time. Mouse survival was evaluated beginning from the transplantation of MDA-MB-231-Luc breast cancer cells and continuing until death. Notably, mice treated with PTX-PLGA nanofibrous membranes exhibited the longest survival duration (Figure 9). Collectively, these results provide evidence that PLGA-PTX treatment reduced in vivo tumor growth and metastasis and contributed to prolonged survival.

#### 3.2.5. In Vivo Pharmacokinetics of PTX

**Figure 10:**
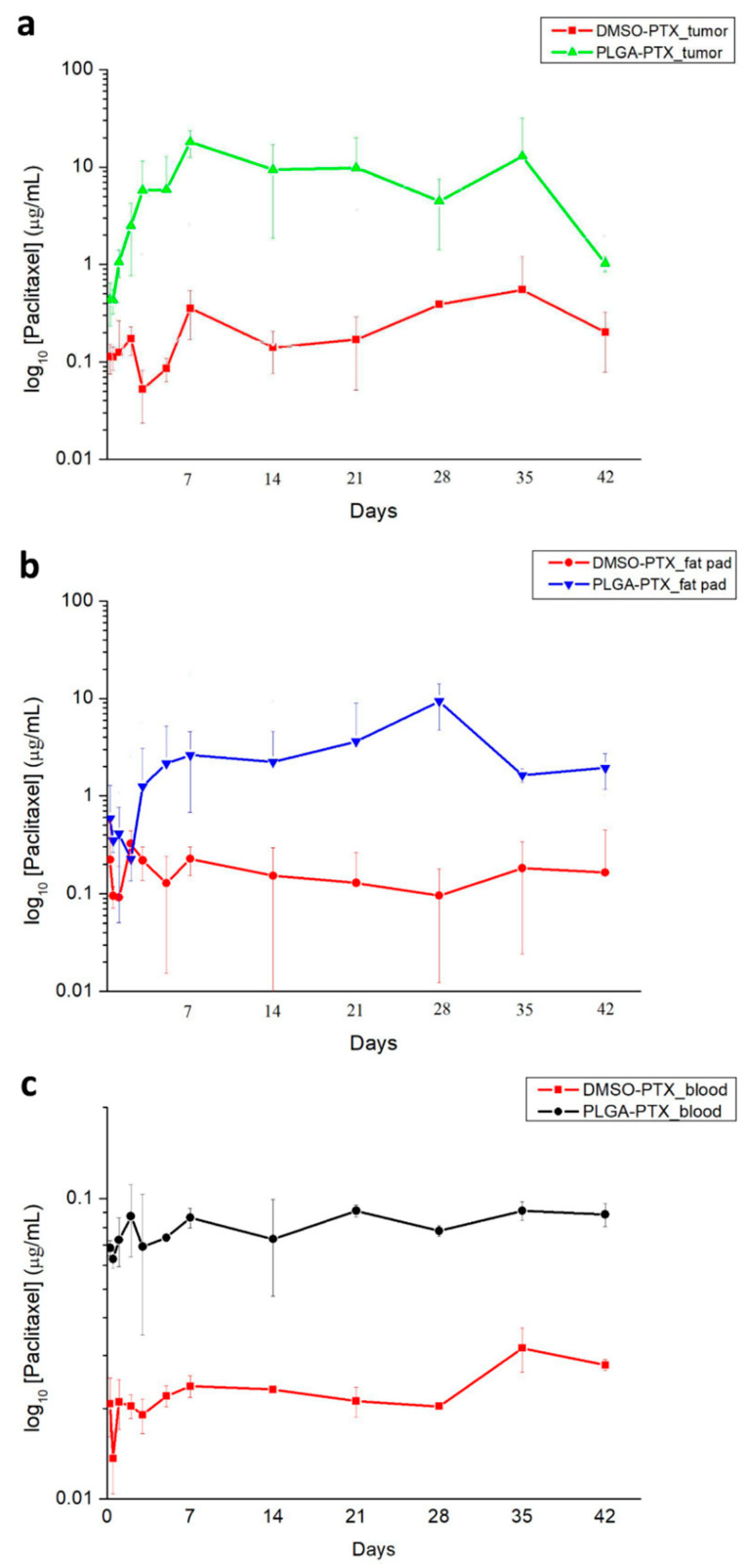
PTX concentrations in the tumor, fat pad, and peripheral blood. PTX was not detectable in DMSO-Ctrl and PLGA-Ctrl groups. PTX concentrations in the tumor (**a**) and fat pad (**b**) were much higher in the PLGA-PTX group than in the DMSO-PTX group from day 3 to day 42. (**c**) Plasma PTX concentrations. The PTX concentration was consistently higher in the PLGA-PTX group than in the DMSO-PTX group throughout the 42-day experiment.

The in vivo drug release behavior was characterized for 6 weeks at 12 time points. The measured concentrations of PTX in the tumor, fat pad, and peripheral blood are shown in Figure 10. PTX concentrations in the tumor and fat pad were consistently greater in the PLGA-PTX group than in the DMSO-PTX group from day 3 to the end of the 42-day experiment (Figure 10a,b). The PTX concentration in the plasma (Figure 10c) was also consistently higher in the PLGA-PTX group, thus confirming the greater inhibitory effect of PLGA-PTX nanofibers on systemic metastasis compared with the DMSO-PTX treatment.

## 4. Discussion

Recent studies have emphasized the importance of the tumor microenvironment for regulating metastasis [34]. Based on the concept of premetastatic niches, primary tumors likely contribute to the formation of microenvironments in distant organs as favorable receptors for metastasizing cells [35,36]. In this study, electrospun PTX-loaded nanofibers can provide locally high drug concentrations to enhance primary tumor control. The strategy of sustained and prolonged release of PTX from nanofibrous membranes provides the critical therapeutic benefits of reducing site-specific tumor growth and effectively inhibiting metastatic TNBC throughout the body.

The experimental results also showed that, compared with pure PTX, PTX nanofibers are more effective in restraining the growth of breast cancer at the primary site. The primary tumor weight at the 4th mammary gland exhibited substantial suppression related to the continuous release of PTX from nanofibers. PTX-loaded PLGA nanofibers also significantly inhibited the proliferation of cancer cells at distant metastatic locations, including the bone, pancreas, lung, liver, and kidney, compared with DMSO-PTX and the control condition. The successful hindrance of metastatic tumor cells by PTX-loaded nanofibers likely resulted from direct cytotoxic effects to distant metastasis, as well as changes in the tumor microenvironment caused by suppression of the primary tumor [10,37,38].

The uniform and prolonged release characteristic of drug-eluting nanofibers is compatible with the MCT strategy. MCT is a multitargeted therapy, which includes stimulation of the antitumor immune response [39] and prevention of stromal activation [40]. Changing the duration of exposure to PTX is an important factor in the effective treatment of human tumors. Raymond et al. [41] reported that both short-term (1 h) and prolonged (14 days) exposure to PTX significantly affected the inhibition of breast cancer cells; long-term exposure may further improve the antitumor activity of PTX. Metronomic PTX chemotherapy has also been associated with a high pathological complete response rate and low toxicity in TNBC patients [42]. The let-7f inhibition and upregulation of antiangiogenic factor thrombospondin-1 may be responsible for the molecular mechanism of cancer cell inhibition [43]. Angiogenesis is considered an essential step for breast cancer progression and dissemination [44]. PTX is a microtubule inhibitor that also possesses antiangiogenic properties involving the downregulation of vascular endothelial growth factor [45]. We found a prominent proliferation of blood vessels on the tumor surface in the control groups but observed only minimal neovascularization in the PLGA-PTX group. The site-specific PLGA-PTX nanofibrous membranes provided locally higher drug concentrations compared with IP PTX injection; this more effectively constrained the neovascularization and mitotic arrest of cancer cells, thereby significantly reducing tumor growth.

Advanced cancer and metastasis are usually associated with cachexia wasting syndrome, characterized by continuous lean mass wasting and weight loss [46]. The estimated prevalence of cachexia is around 40% in patients with breast cancer [47]. Weight loss was observed in the control groups after 28 days with the progression of metastatic disease (Figure 6c). Although the DMSO-PTX group had a less advanced metastatic tumor burden, it exhibited greater weight loss compared with the control groups, presumably due to the systemic toxicity of PTX. In contrast, the PLGA-PTX group showed the best tumor control and continuously increased body weight, which demonstrated that the PTX-loaded nanofibers could effectively enhance the therapeutic effect with minimal complications. The toxicity profile of PTX appears to be both dose- and schedule-dependent; the reduced drug interval of PTX may improve its therapeutic effects and produce a lower toxicity profile [48,49].

In vitro experiments (Figure 4) indicated that the PTX was continuously released from nanofibers and reached approximately 60.73% of cumulative drug release in 6 weeks, which is consistent with the in vivo tumor inhibition findings in the PLGA-PTX group. Notably, from the in vivo drug release curve, the drug concentrations of various tissues (e.g., tumor, fat pad, and circulating blood) were higher in the PLGA-PTX group than in the DMSO-PTX group; this was consistent with the finding that PTX nanofibers have a better tumor suppression effect. The plasma clearance of intravenous PTX is biphasic, such that the first rapid decline occurs within hours due to drug redistribution and elimination, followed by an extended elimination half-life [50,51]. Pharmacokinetic studies in animal models with IP-applied PTX have also shown rapid declines of both plasma and peritoneal concentrations within 24 h [52]. These findings probably explain the absence of a prominent peak plasma concentration in the DMSO-PTX group. Instead, a higher tissue concentration was maintained by the continuous release pattern of PTX. With the continuous PTX release from nanofibers, the PTX concentration increased in both the tissue and plasma compared with the weekly IP infusion; this contributed to an improved antitumor effect and prolonged survival.

There were a few limitations in this study. First, the PTX solution was mixed in DMSO and administered to the mice via IP injection. The absorption and distribution of PTX in mice may be different from these characteristics in humans, thus causing treatment efficacy bias. Second, in patients with metastatic disease who have undergone previous resection of the primary tumor, PTX-loaded nanofibers may have different effects. Further studies are warranted for detailed investigation. Third, we used nanofibers with a 10:1 polymer-to-drug ratio for better hydrophilicity and more prolonged drug release. Changes in the composition and release profiles of drug-loaded nanofibers may lead to different results in future studies. Fourth, immune reactions to the PLGA membrane and the tumor could not be fully investigated because this study used immunosuppressed mice. The final limitation lies in that there was only one model used in this study. Xenografts from cancer cell lines are not the best models for preclinical development of therapeutic drugs. Patient-derived xenografts should be completed. All these will be the topics of our future studies.

Compared to nanoparticles, nanofibers are rarely used for treating metastasis [53,54,55]. In fact, drug-loaded nanofibers can allow systemic drug release to control metastasis, while effectively increasing the site-specific local drug concentration for primary tumor control. In addition to target therapy and chemotherapy, several promising treatment options (e.g., immune checkpoint therapy, epidermal growth factor receptor inhibitors, and androgen receptor inhibitors) are under active clinical investigation for TNBC. Combined chemotherapy with an immune checkpoint inhibitor or other novel therapies may be helpful [56,57,58]. The use of coaxial electrospinning to create nanofibers can facilitate the administration of multiple drugs for sequential delivery applications [59]. Combination therapy using nanofibers as the vector may provide additional benefits for future cancer treatment and deserves further investigation.

## 5. Conclusions

PTX is a standard drug for the treatment of metastatic TNBC. To maximize the therapeutic effect of PTX and minimize the side effects, we used PTX-loaded PLGA nanofibers in mice with human TNBC cells, which revealed significant inhibition of the primary tumor and distant metastasis compared with control groups. Furthermore, the PTX-loaded PLGA group showed a continuous and slow increase in weight and extended survival, suggesting that the use of PTX-loaded PLGA nanofibers for direct tumor site-specific therapy could reduce systemic toxicity associated with PTX treatment, while enhancing its antitumor efficacy. Through the uniform and sustained release of PTX from nanofibers, the MCT strategy successfully improves the efficacy of PTX compared with conventional chemotherapy and could be a promising option for future cancer treatment.

## Data Availability

The data used to support the findings of this study are included within the article.

## Supplementary Materials

The following are available online at https://www.mdpi.com/article/10.3390/cancers13133350/s1, Figure S1: Uncropped blots used in Figure 8c.
