# Supplementary material for: Enhanced Paclitaxel Efficacy to Suppress Triple-Negative Breast Cancer Progression Using Metronomic Chemotherapy with a Controlled Release System of Electrospun Poly-d-l-Lactide-Co-Glycolide (PLGA) Nanofibers
Source: Cancers (Basel). 2021 Jul 3;13(13):3350. doi: 10.3390/cancers13133350 (PMC8268060; doi:10.3390/cancers13133350)
Supplement: Supplementary file 1 [file cancers-13-03350-s001.zip › cancers-1267517-supplementary.pdf]

Supplemental Material

Enhanced Paclitaxel Efficacy to Suppress Triple-Negative Breast Cancer Progression Using Metronomic Chemotherapy with a Controlled Release System of Electrospun Poly-D-L-Lactide-Co-Glycolide (PLGA) Nanofibers

Ming-Yi Hsu, Cheng-Hsien Hsieh, Yu-Ting Huang, Sung-Yu Chu, Chien-Ming Chen, Wei-Jiunn Lee and Shih-Jung Liu

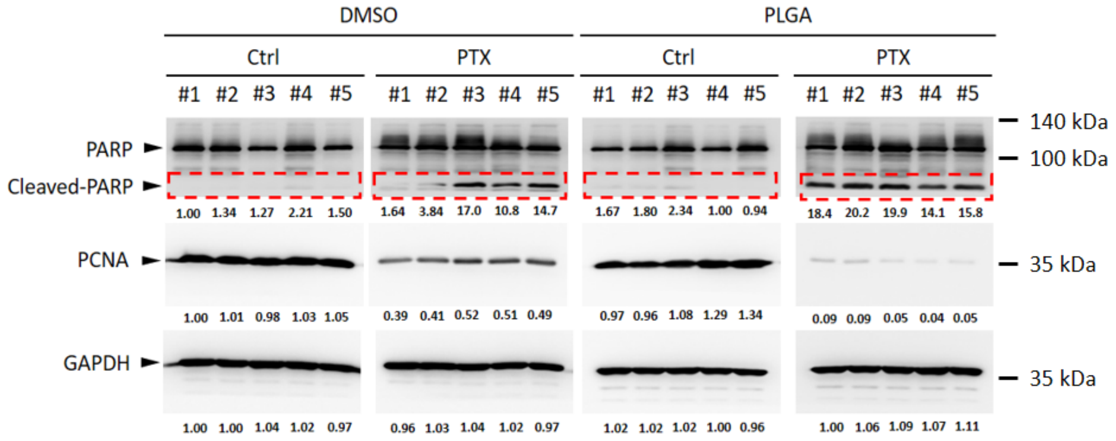

Figure S1. Uncropped blots used in Figure 8c. Representative western blot analysis of PARP, PCNA, and GAPDH in each treatment group of the tumor samples. Quantitative results of indicated protein levels, which were adjusted to the GAPDH protein level.
